# A DNA-damage immune response assay combined with PET biomarkers predicts response to neo-adjuvant chemotherapy and survival in oesophageal adenocarcinoma

**DOI:** 10.1038/s41598-021-92545-w

**Published:** 2021-06-22

**Authors:** Kieran G. Foley, Anita Lavery, Eoin Napier, David Campbell, Martin M. Eatock, Richard D. Kennedy, Kevin M. Bradley, Richard C. Turkington

**Affiliations:** 1 Velindre Cancer Centre, Cardiff, UK; 2 Queen’s University Belfast, Belfast, UK; 3 Belfast Health and Social Care Trust, Belfast, UK; 4 Almac Diagnostics, Craigavon, UK; 5 Wales Research & Diagnostic Positron Emission Tomography Imaging Centre (PETIC), Cardiff University, Cardiff, UK

**Keywords:** Cancer, Biomarkers, Gastroenterology, Oncology

## Abstract

18F-fluorodeoxyglucose PET-CT may guide treatment decisions in patients with oesophageal adenocarcinoma (OAC). This study evaluated the added value of maximum standardised uptake value (SUVmax) to a novel DNA-damage immune response (DDIR) assay to improve pathological response prediction. The diagnostic accuracy of PET response and the prognostic significance of PET metrics for recurrence-free survival (RFS) and overall survival (OS) were assessed. This was a retrospective, single-centre study of OAC patients treated with neo-adjuvant chemotherapy from 2003 to 2014. SUVmax was recorded from baseline and repeat PET-CT after completion of pre-operative chemotherapy. Logistic regression models tested the additional predictive value of PET metrics combined with the DDIR assay for pathological response. Cox regression models tested the prognostic significance of PET metrics for RFS and OS. In total, 113 patients were included; 25 (22.1%) were DDIR positive and 88 (77.9%) were DDIR negative. 69 (61.1%) were PET responders (SUVmax reduction of 35%) and 44 (38.9%) were PET non-responders. After adding PET metrics to DDIR status, post-chemotherapy SUVmax (hazard ratio (HR) 0.75, p = 0.02), SUVmax change (HR 1.04, p = 0.003) and an optimum SUVmax reduction of 46.5% (HR 4.36, p = 0.021) showed additional value for predicting pathological response. The optimised SUVmax threshold was independently significant for RFS (HR 0.47, 95% CI 0.26–0.85, p = 0.012) and OS (HR 0.51, 95% CI 0.26–0.99, p = 0.047). This study demonstrated the additional value of PET metrics, when combined with a novel DDIR assay, to predict pathological response in OAC patients treated with neo-adjuvant chemotherapy. Furthermore, an optimised SUVmax reduction threshold for pathological response was calculated and was independently significant for RFS and OS.

## Introduction

Despite advances in surgical and oncological management, the prognosis of patients with oesophageal adenocarcinoma (OAC) remains poor^1^. The incidence of OAC is rising in the Western world^2^, but the majority of patients still present with advanced disease and palliation is the only treatment available to them.

Neo-adjuvant chemotherapy is currently standard of care in the UK for the minority deemed potentially curable with major surgery^3^. However, only 15% of OAC patients exhibit a meaningful pathological response^4^, precipitating unnecessary surgical delays in the majority, in which time disease can progress, and patients can become physiologically deconditioned before their operation. The majority of these patients then relapse and eventually succumb to their disease^5^. Improving the selection of patients who are likely to respond to neo-adjuvant chemotherapy is critical. There is a pressing need to identify biomarkers capable of predicting response to neo-adjuvant chemotherapy to facilitate optimal patient selection prior to surgical resection.

New discoveries have identified genomic sub-types of OAC, one of which demonstrates deficiency in DNA damage repair in 20%^6^. Recently, the accuracy of a novel DNA-damage immune response (DDIR) assay to predict pathological responders to DNA-damaging platinum-based chemotherapy was evaluated^7^. Patients with a positive DDIR assay had a significantly higher pathological response rate (p = 0.033) and were associated with improved recurrence-free survival (RFS, p = 0.042) and overall survival (OS, p = 0.015) in multi-variable analysis. These early results are promising, but the prediction of patients likely to respond to neo-adjuvant chemotherapy could, and must, be improved further.

Positron emission tomography (PET) combined with computed tomography (PET-CT) is now routinely used in the OAC staging pathway^8^. PET-CT improves the sensitivity for the detection of distant metastases compared to contrast-enhanced CT from 52 to 71%^9^ and changes management in 20–40% of patients^10^. Metabolic response on PET-CT has also been shown to predict pathological response^11^, therefore imaging biomarkers, in combination with the DDIR assay may further characterise the tumour microenvironment (TME) and predict which patients will respond.

In this current study, we hypothesised that the addition of PET imaging parameters to clinical parameters and the DDIR assay would further improve the prediction of pathological response to neo-adjuvant chemotherapy. We aimed to assess the diagnostic accuracy of PET response in this cohort and calculate the prognostic significance of PET metrics for RFS and OS.

## Materials and methods

### Patient cohort

This retrospective, single-centre study included patients with oesophageal adenocarcinoma who were treated with neo-adjuvant platinum-based chemotherapy and resection between 2003 and 2014. This current study builds on the investigation of a DDIR assay in this patient cohort^7^. The study was performed according to the Transparent Reporting of a multivariable prediction model for Individual Prognosis or Diagnosis (TRIPOD) guidance^12^. Ethical approval was obtained for biological sample collection and analysis in conjunction with detailed clinical annotation from the Northern Ireland Biobank (NIB12-0032) and the Office for Research Ethics Committees Northern Ireland (ORECNI: 13/NI/0149). All research was performed in accordance with relevant guidelines, regulations and the Declaration of Helsinki, and informed consent was obtained from all participants.

### Clinical variables

All patients had biopsy-proven adenocarcinoma of the oesophagus or gastro-oesophageal junction following upper gastrointestinal (GI) endoscopy. Clinical variables including age at diagnosis, gender, radiological staging, pathological staging, neo-adjuvant chemotherapy regimen and outcome were recorded.

### PET-CT protocol

Two PET-CT scanners and protocols were used during the study period. All patients had pre- and post-treatment PET-CT imaging using the same scanner. Before December 2011, PET-CT was performed using a GE Discovery LS 2-dimensional (2D) scanner, without a time-of-flight (TOF) algorithm. Patients received a dose of 375 MBq of 18F-FDG. After December 2011, a GE Discovery 690 3D scanner with TOF algorithm was used. Patients received a 18F-FDG dose of 3.5 MBq/Kg. SUVmax was recorded by the reporting PET-CT radiologist and documented in the corresponding report.

### Radiological staging

**Figure 1:**
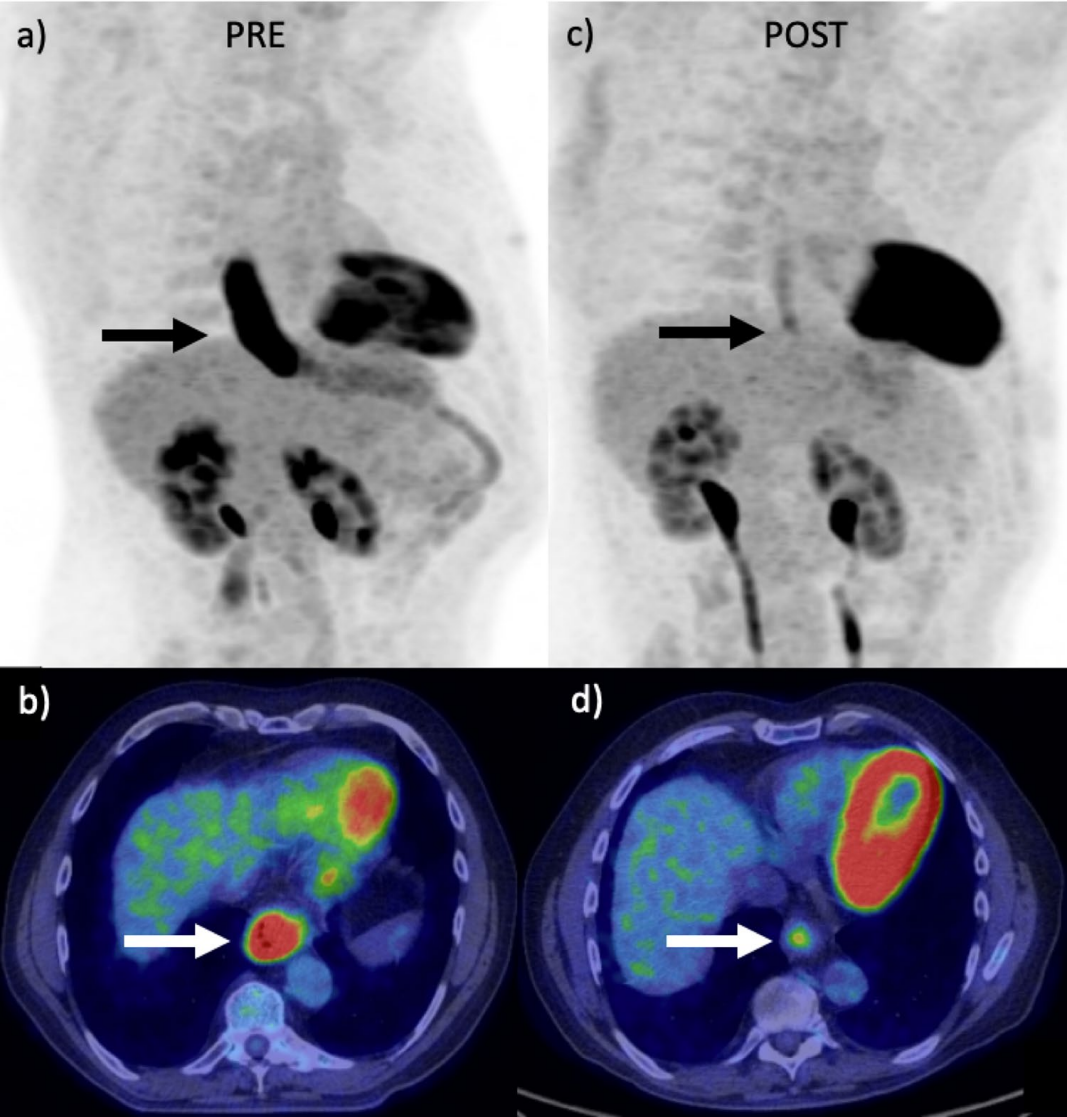
Selected images from a patient with a distal oesophageal adenocarcinoma. Maximum intensity projection (MIP) (**a**) and fused axial PET-CT images (**b**) demonstrated a large FDG-avid tumour. After neo-adjuvant chemotherapy was completed, a repeat PET-CT (**c**, **d**) showed there had been an excellent metabolic response. This patient had a positive DDIR assay and final pathological examination indicated a good response (tumour regression grade 2).

Radiological staging followed international guidelines and consisted of initial contrast-enhanced CT, following by PET-CT + /− EUS for more detailed staging^8^. All patients were staged according to the contemporaneous International Union for Cancer Control (UICC) Tumour Node Metastasis (TNM) classification (7th edition). Three PET metrics were retrospectively recorded from the PET-CT examination reports. Baseline SUVmax represented the highest SUV in the primary tumour prior to treatment initiation. Post neo-adjuvant treatment SUVmax was recorded after chemotherapy completion and prior to surgery. The second PET-CT was performed to re-stage patients prior to oesophagectomy to detect interval metastases^13^. The change in SUVmax between examinations was calculated by subtracting the post-treatment SUVmax from the baseline SUVmax. A PET response was defined as a reduction in SUVmax of 35% between baseline and post-treatment PET-CT^11^ (Fig. 1).

### Treatment pathway

Patients had 12 weeks of neo-adjuvant platinum-based chemotherapy equalling three cycles of either epirubicin, cisplatin and capecitabine (ECX), or epirubicin, cisplatin and 5-fluorouracil (ECF). The time between the last cycle of chemotherapy finishing and the second PET-CT was 21 days. Surgical resection was then performed 4–6 weeks after completing neo-adjuvant chemotherapy.

### Pathological staging

Pathological resection specimens were reported following the minimum recommended dataset^14^. The TNM classification was assigned, and the degree of adenocarcinoma differentiation, local vascular invasion (LVI) and circumferential resection margin (CRM) involvement were categorised. Pathological response was assigned using the Mandard classification^15^, with responders classified as tumour regression grade (TRG) 1–2, and non-responders as TRG 3–5^4^. The details of the DDIR assay are found in Turkington et al.^7^.

### Outcome data

Data on three clinically important outcomes were collected; pathological response, RFS and OS. RFS was defined as the time from surgical resection to recurrence or death, or date of last follow-up. OS was defined as the time from diagnosis to death from any cause, or date of last follow-up. Dates of each outcome were captured, and the time interval between diagnosis and outcome recorded in days.

### Statistical analysis

Statistical analysis was performed using R software (version 3.6.1)^16^. Cases with missing data were excluded. Correction for multiple comparisons was performed using the Bonferroni method^17^. Differences between continuous and categorical variables were tested with Mann–Whitney U tests and chi-square tests, respectively. Diagnostic performance of PET metrics was calculated with sensitivity, specificity, positive predictive value (PPV) and negative predictive value (NPV). Receiver operator characteristic (ROC) curves and Youden method were used to calculate optimum thresholds of continuous variables for diagnostic performance. Univariable analysis tested each variable for association with RFS and OS. A series of multi-variable regression models were constructed to analyse the additional value of the DDIR assay and PET metrics to predict pathological response, RFS and OS.

## Results

### Clinicopathological and radiological characteristics of the cohort

**Figure 2:**
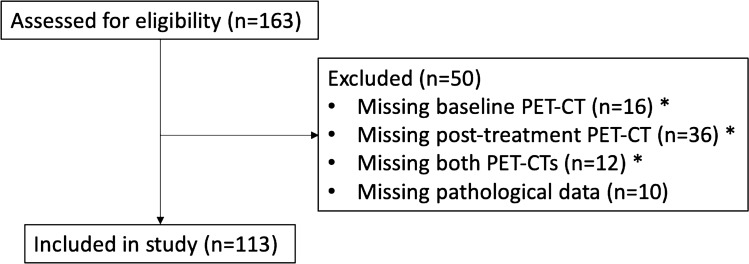
A CONSORT study flow diagram detailing the inclusion of patients in the study. The total patients excluded for missing PET-CT data were 40.

In total, 163 patients were considered for inclusion. After selection criteria were applied, 113 patients were analysed after cases with missing PET (n = 40) and TRG data (n = 10) were excluded. Table 1 details the characteristics of the cohort. A CONSORT study flow diagram is shown in Fig. 2.

**Table 1:** Baseline characteristics of patient cohort.

	DDIR negative (n = 88)	DDIR positive (n = 25)	p-value
Age (years) median (range, IQR)	63.00 (28.00–83.00, 9.25)	62.00 (47.00–78.00, 11.00)	0.88
**Gender**			
Male	66 (75.00%)	22 (88.00%)	0.27
Female	22 (25.00%)	3 (12.00%)	
**Chemotherapy regimen**			
ECX	60 (68.18%)	18 (72.00%)	0.64
ECF	25 (28.41%)	7 (28.00%)	
ECF/ECX	3 (3.41%)	0 (0.00%)	
**cT-stage**			
T1/2	10 (11.36%)	6 (24.00%)	0.19
T3/4a	72 (81.82%)	17 (68.00%)	
TX	6 (6.80%)	2 (8.00%)	
**cN-stage**			
N0	23 (26.14%)	5 (20.00%)	0.73
N+	52 (59.09%)	16 (64.00%)	
NX	13 (14.80%)	4 (16.00%)	
**cM-stage**			
M0	88 (100.00%)	25 (100.00%)	N/A
**pT-stage**			
pCR/T1/2	26 (29.55%)	14 (56.00%)	0.028
T3/4a	62 (70.45%)	11 (44.00%)	
**pN-stage**			
N0	34 (38.64%)	13 (52.00%)	0.33
N+ (N1–3)	54 (61.36%)	12 (48.00%)	
**pM-stage**			
M0	88 (100.00%)	25 (100.00%)	N/A
**Degree of differentiation**			
Well	3 (3.41%)	3 (12.00%)	0.20
Moderate	33 (37.50%)	8 (32.00%)	
Poor	51 (57.95%)	12 (48.00%)	
Missing	1 (1.10%)	2 (8.00%)	
**LVI**			
Negative	34 (38.64%)	9 (36.00%)	1.00
Positive	53 (60.23%)	15 (60.00%)	
Missing	1 (1.10%)	1 (4.00%)	
**CRM**			
R0	43 (48.86%)	19 (76.00%)	0.029
R1	45 (51.14%)	6 (24.00%)	
**Tumour regression grade**			
TRG 1	2 (2.27%)	4 (16.00%)	0.025
TRG 2	7 (7.95%)	0 (0.00%)	
TRG 3	18 (20.45%)	3 (12.00%)	
TRG 4	46 (52.27%)	11 (44.00%)	
TRG 5	15 (17.05%)	7 (28.00%)	
**PET response**			
Non-responder	35 (39.77%)	9 (36.00%)	0.91
Responder	53 (60.23%)	16 (64.00%)	
**Pathological response**			
Non-responder	79 (89.77%)	21 (84.00%)	0.66
Responder	9 (10.23%)	4 (16.00%)	
**Recurrence**			
No	38 (43.18%)	16 (64.00%)	0.11
Yes	50 (56.82%)	9 (36.00%)	

*ECX* epirubicin, cisplatin and capecitabine, *ECF* epirubicin, cisplatin and 5-fluorouracil; *c* clinical, *p* pathological, *TX* T-stage not assessed, *NX* N-stage not assessed, *GX* grade of differentiation not assessed, *pCR* complete pathological response, *N/A* not applicable, *LVI* local vascular invasion, *CRM* circumferential resection margin, *TRG* tumour regression grade.

Post-treatment PET-CT was performed after completion of chemotherapy approximately 15 weeks after baseline PET/CT. Surgery was performed up to 6 weeks from post-treatment PET-CT. Summary statistics of SUVmax distribution between DDIR negative and positive tumours are detailed in Supplementary Table S1. No significant differences in SUVmax (p = 0.47), post-NACT SUVmax (p = 0.52), or change in SUVmax (p = 0.21) were found between the two PET-CT scanners. Histograms and waterfall plots of SUVmax distributions are available in Supplementary Figs. S1–S3.

### Association of clinicopathological and radiological variables with DDIR status, pathological response and recurrence

There were several significant associations between clinical variables and clinical outcomes. Notably, pT-stage was associated with pathological response (p < 0.001), and pN-stage and CRM were associated with recurrence (p < 0.001, respectively) (Supplementary Table S2).

**Figure 3:**
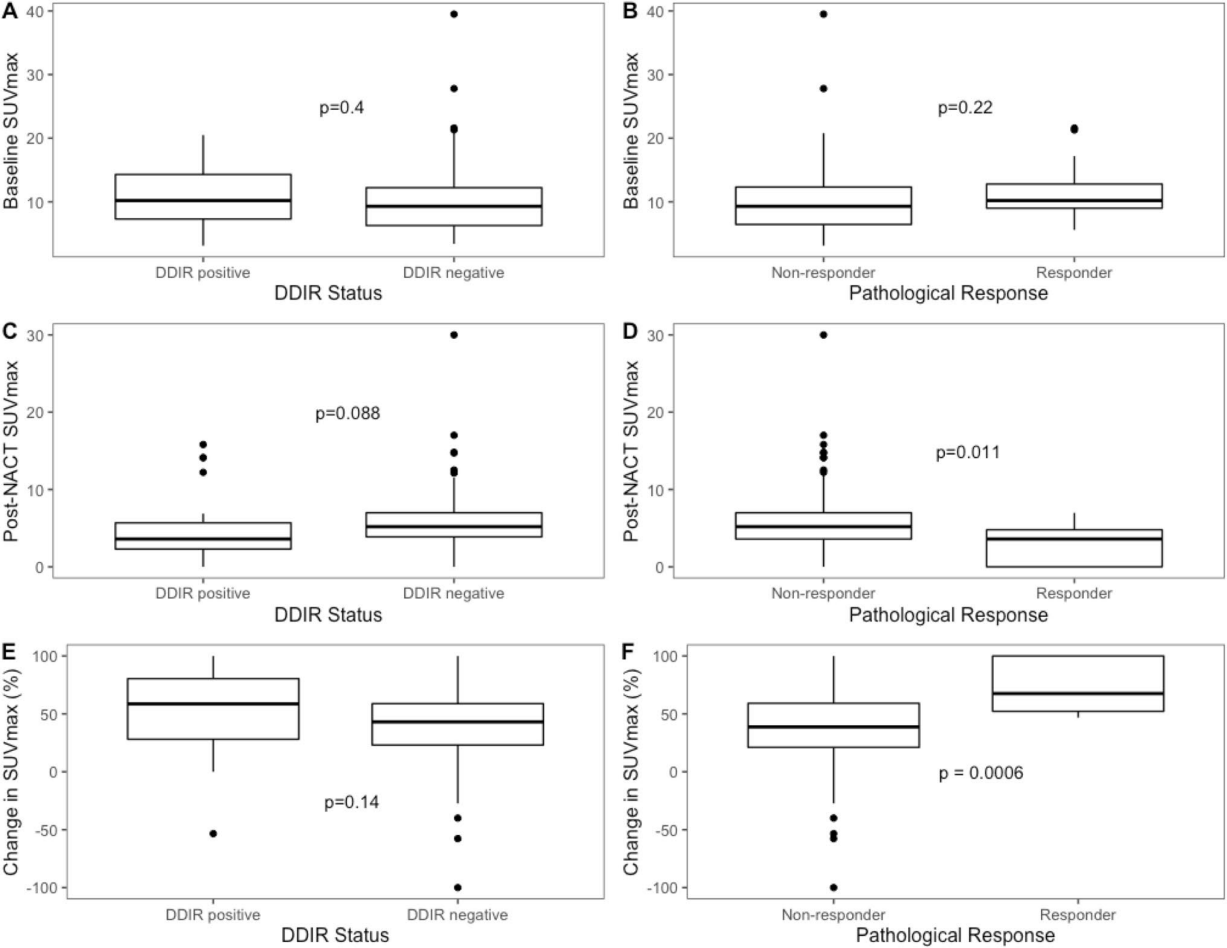
Boxplots of differences between baseline, post neo-adjuvant chemotherapy and change in SUVmax for DDIR status and pathological response (unadjusted p-values shown within boxplots).

Boxplots showing the differences in PET metrics between DDIR status and pathological response status are shown in Fig. 3. There were significant associations between post-NACT SUVmax (p = 0.03) and change in SUVmax (p = 0.002) with pathological response after adjusting for multiple comparisons (Table 2).

**Table 2:** Association of PET metrics with DDIR status, pathological response and recurrence.

	U-statistic	Unadjusted p-value	Adjusted p-value
**DDIR status**			
Baseline SUVmax	979.00	0.40	1.00
Post-NACT SUVmax	1347.00	0.09	0.26
Change in SUVmax	885.50	0.14	0.42
**Pathological response**			
Baseline SUVmax	513.00	0.22	0.66
Post-NACT SUVmax	934.50	0.01	0.03
Change in SUVmax	268.50	0.0006	0.002
**Recurrence**			
Baseline SUVmax	1842.00	0.15	0.46
Post-NACT SUVmax	1593.00	1.00	1.00
Change in SUVmax	1690.50	0.58	1.00

### Sensitivity and specificity of PET response to predict pathological response

In the entire cohort, the sensitivity and specificity of the 35% SUVmax reduction threshold to predict pathological response were 1.00 and 0.44, respectively. The optimal SUVmax reduction threshold for the best sensitivity and specificity values to predict pathological response were calculated with the Youden method. Cases of progression (defined as increase in SUVmax between PET-CT examinations) were excluded for this purpose. Using a threshold of 46.5%, the optimum sensitivity and specificity was 0.69 and 0.66, respectively. This threshold produced a c-statistic of 0.78. (Supplementary Fig. S4) Table 3 shows the diagnostic accuracy results for the DDIR status, DDIR status combined with a PET threshold of 35%, and DDIR status when combined with the optimised 46.5% threshold. Combining PET metrics with DDIR status improved the specificity for predicting pathological response up to 0.90 without loss of sensitivity.

**Table 3:** Sensitivity, specificity, positive predictive value and negative predictive value for pathological response of DDIR Status, DDIR combined with a PET Threshold of 35% and with the optimised SUVmax reduction threshold.

	DDIR status		DDIR status and PET threshold (35%)		DDIR status and optimised threshold (46.5%)	
	Value	95% CI	Value	95% CI	Value	95% CI
Sensitivity	0.31	0.09–0.61	0.31	0.09–0.61	0.31	0.09–0.61
Specificity	0.79	0.70–0.87	0.88	0.80–0.94	0.90	0.82–0.95
Positive predictive value	0.16	0.05–0.36	0.25	0.07–0.52	0.29	0.08–0.58
Negative predictive value	0.90	0.81–0.95	0.91	0.83–0.96	0.91	0.83–0.96

Optimum SUVmax reduction thresholds for clinical outcomes (pathological response and recurrence) and individual ROC curves for each PET metric are included in Supplementary Table S3 and Supplementary Fig. S5.

### Combined DDIR status and PET metrics predict pathological response better than either metric alone

Having demonstrated the association of PET metrics with pathological response, we sought to integrate this information with DDIR status to assess the performance of a combined assay. Two significant associations were demonstrated on uni-variable analysis after Bonferroni correction. Change in SUVmax (p = 0.029) and pT-stage (< 0.001) were both significantly associated with pathological response. DDIR status (unadjusted p = 0.428) was not associated with pathological response.

In terms of PET response, logistic regression was not reliable (unadjusted p = 0.991) because the sensitivity of PET response at the 35% threshold was 100%, therefore no false negative results were recorded. Therefore, this variable was omitted from further analysis predicting pathological response. Full details of the univariable analysis are included in Supplementary Table S4.

On multi-variable analysis, post-NACT SUVmax (p = 0.020), change in SUVmax (p = 0.003), and PET response at optimised 46.5% threshold (p = 0.021) were independently and significantly associated with pathological response. (Table 4) In contrast to Turkington et al.^7^, DDIR status was not significantly associated with pathological response. This result may be explained by the analysis of a sub-group from the original cohort which included relatively few pathological responses. Furthermore, the AIC values of the logistic regression models indicate that the models combining DDIR and PET metrics performed better than DDIR status alone.

**Table 4:** Results for each multi-variable model to predict pathological response.

Model	Variable	HR	SE	p-value	AIC
1	DDIR	1.67	0.65	0.428	84.07
2	DDIR	1.65	0.65	0.443	85.23
	Baseline SUVmax	1.05	0.05	0.339	
3	DDIR	1.21	0.70	0.787	78.8
	Post-NACT SUVmax	0.75	0.12	0.020	
4	DDIR	1.05	0.71	0.951	74.51
	Change in SUVmax	1.04	0.01	0.003	
5	DDIR	1.66	0.67	0.450	80.24
	PET response at optimised 46.5% threshold	4.36	0.64	0.021	

*DDIR* DNA-damage immune response, *NACT* neo-adjuvant chemotherapy, *SUVmax* maximum standardised uptake value, *HR* hazard ratio, *SE* standard error, *AIC* Akaike Information Criterion.

### Survival analysis

The median RFS of all patients was 36.7 months (95% CI 21.1–63.2). The median OS of the cohort was 43.9 months (95% CI 33.2–not reached).

RFS rates at 1, 2, and 5 years were 73.4% (95% CI 65.6–82.0%), 55.4% (95% CI 46.9–65.4%), and 39.6% (95% CI 30.8–51.1%), respectively. OS rates at 1, 2, and 5 years were 78.7% (95% CI 71.5–86.6%), 67.9% (59.7–77.1%), and 46.7% (37.1–58.7%), respectively.

After adjusting for multiple comparisons, seven variables were associated with RFS in uni-variable analysis; pT-stage, pN-stage, LVI, CRM, total positive nodes after resection, positive nodal ratio, and TRG. Similarly, seven variables were associated with OS; pT-stage, pN-stage, LVI, CRM, total positive nodes after resection, positive nodal ratio, and recurrence.

**Figure 4:**
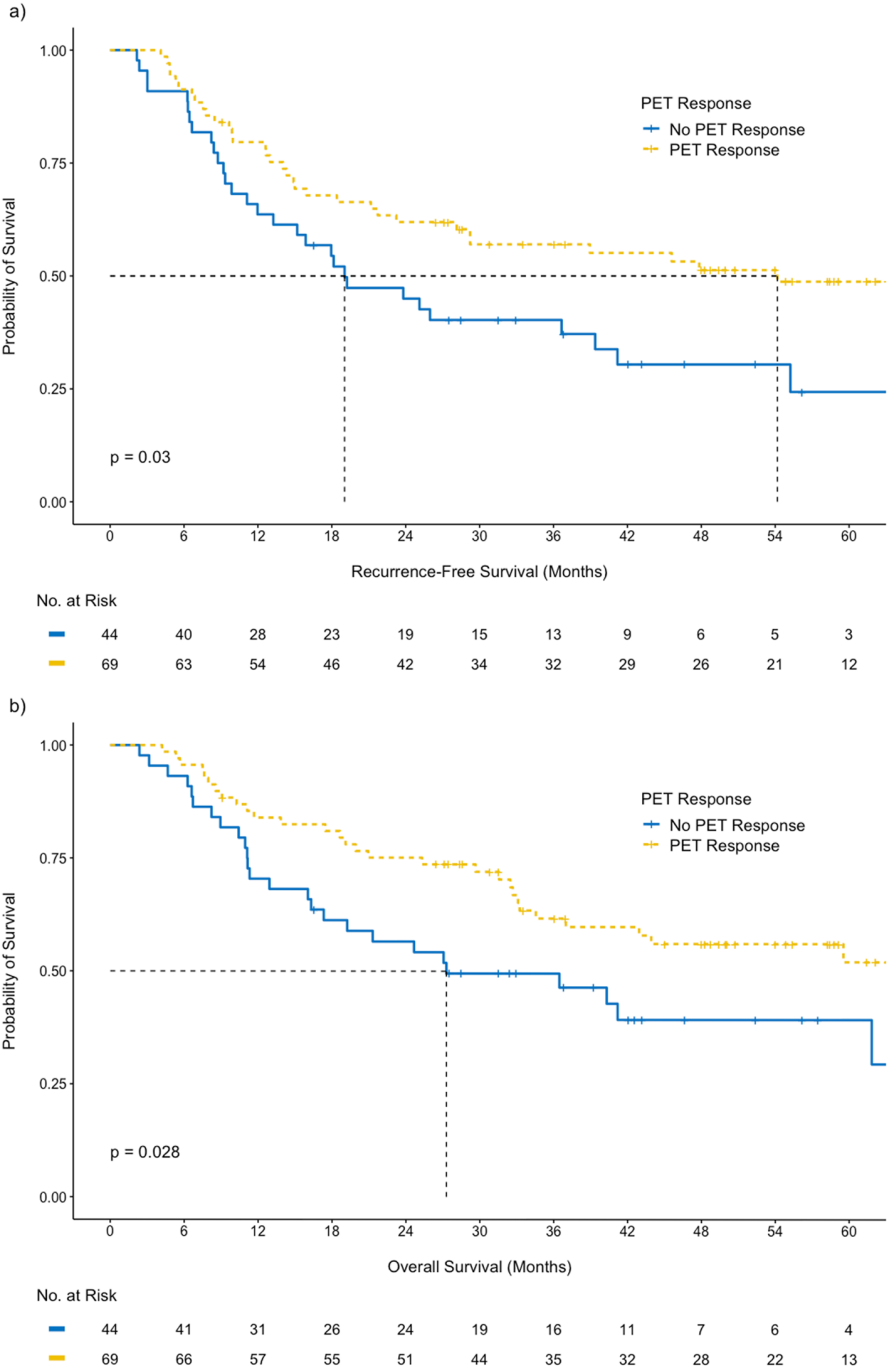
Cumulative (**a**) recurrence-free and (**b**) overall survival depending on PET response status using a 35% SUVmax reduction threshold level.

Full details of the uni-variable analysis results for RFS and OS are included in the Supplementary Tables S5 and S6. There were significant differences in RFS (p = 0.030) and OS (p = 0.028) in PET responders versus non-responders at the 35% reduction threshold. (Fig. 4) Median RFS of PET responders was 54.2 months (95% CI 28.1–not reached) and PET non-responders was 19.0 months (95% CI 13.2–41.2). Median OS of PET responders was not reached, and median OS of PET non-responders was 27.3 months (95% CI 17.3–not reached).

**Figure 5:**
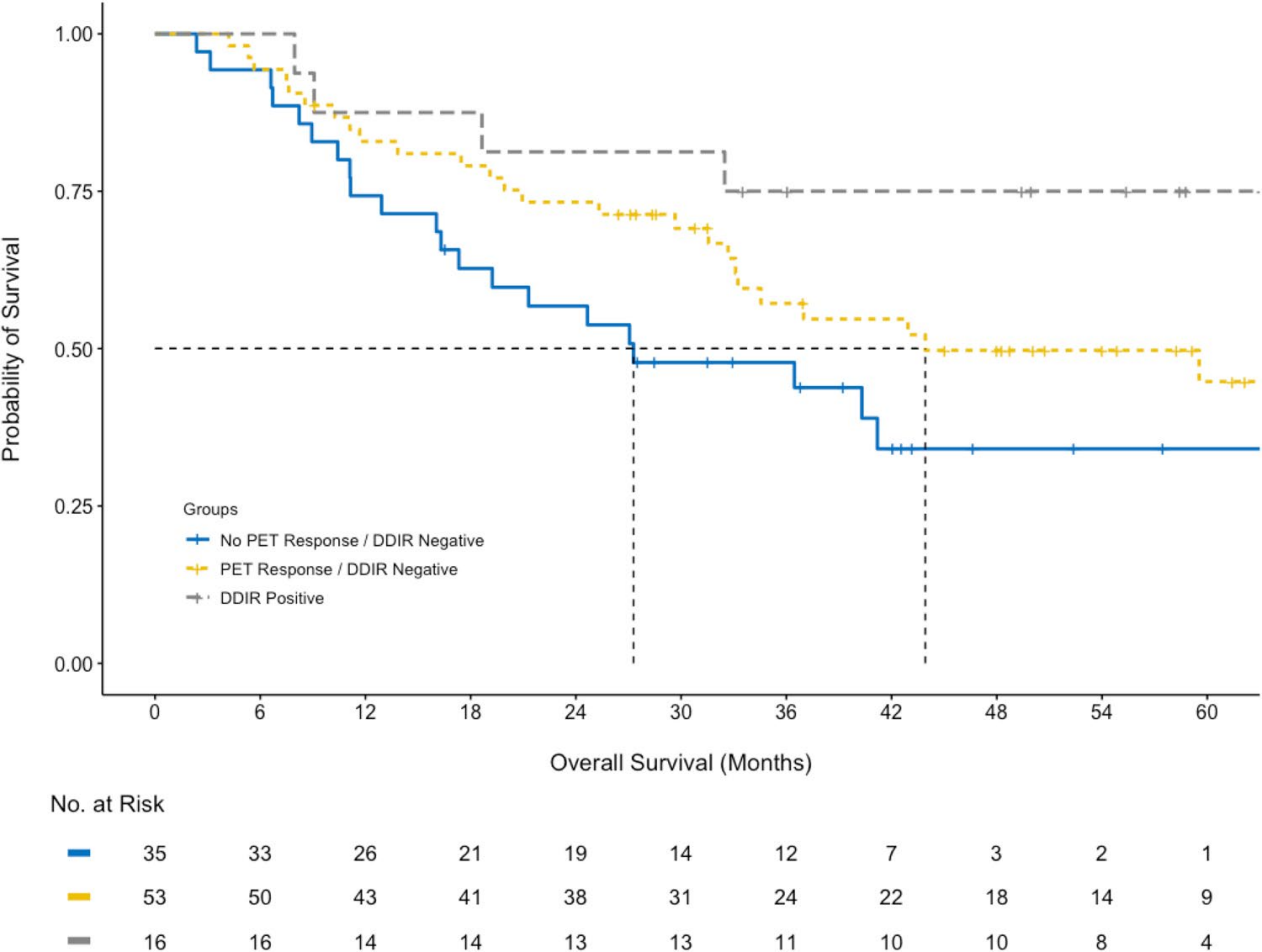
Cumulative overall survival differences between three groups; no PET response and DDIR negative, PET response and DDIR negative, and DDIR positive. The latter group comprised both PET responders and non-responders because only nine patients had no PET response and were DDIR positive.

Three groups were constructed to compare the survival differences between combined DDIR status and PET response; no PET response and DDIR negative, PET response and DDIR negative, and DDIR positive. The latter group comprised both PET responders and non-responders because only nine patients had no PET response and were DDIR positive, so these nine patients were combined with the other DDIR positive patients for this specific analysis. There were significant differences in RFS (p = 0.01) and OS (p = 0.03) (Fig. 5) between groups.

**Figure 6:**
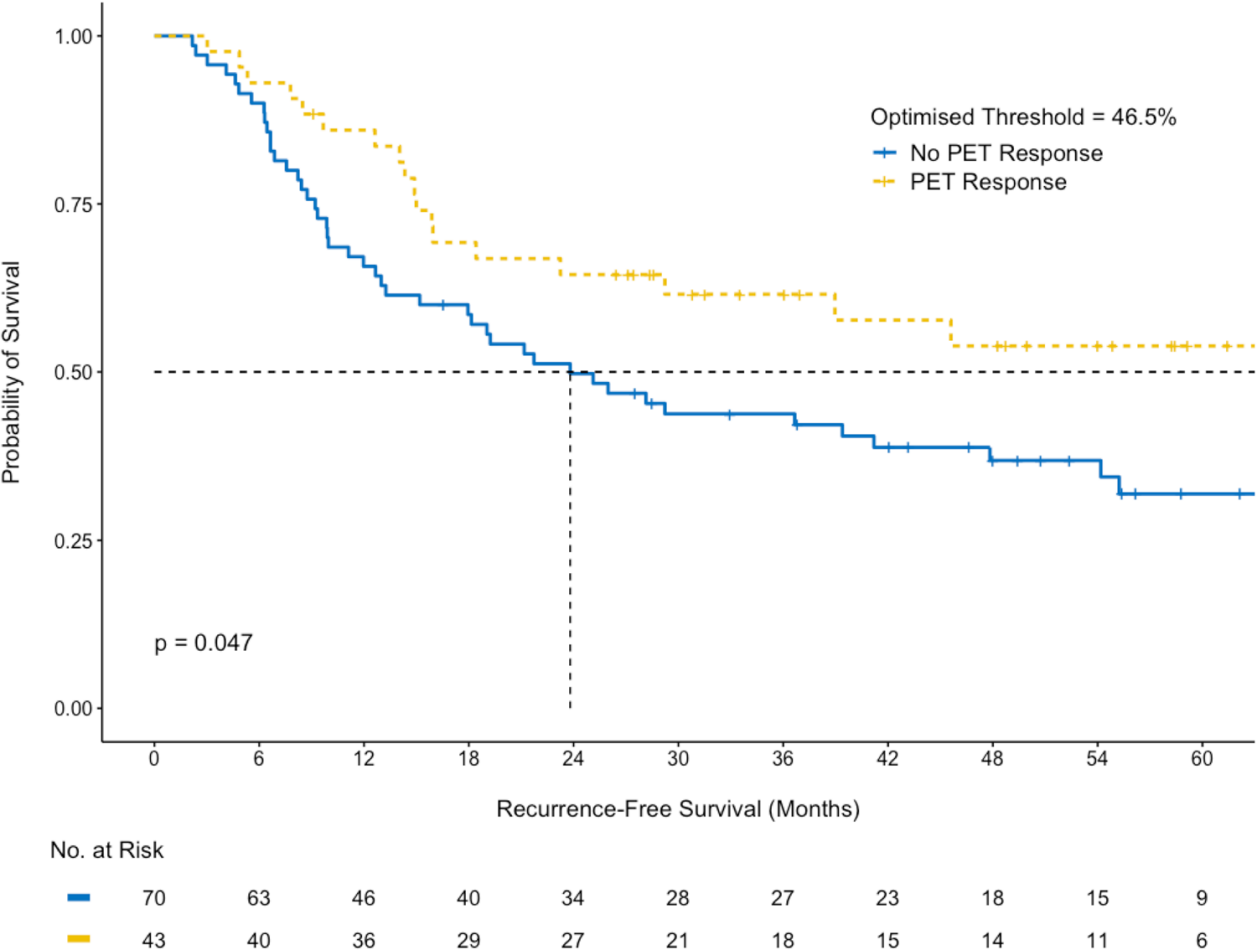
Recurrence-free survival difference between pathological responders and non-responders using the optimised threshold of 46.5% reduction in SUVmax.

The prognostic significance for RFS and OS was also tested using the optimised 46.5% PET response threshold to predict pathological response. There was a significant difference (p = 0.047) in RFS between PET responders (63.2 months; 95% CI 29.2–not reached) and PET non-responders (23.8 months; 95% CI 15.2–54.2) using the 46.5% threshold optimised for pathological response, although the survival difference between the two groups was larger using the 35% SUVmax reduction threshold. (Fig. 6) The optimised 46.5% threshold did not meet statistical significance for OS (p = 0.099; Supplementary Fig. S6). Additional cumulative survival plots for DDIR status and pathological response with RFS and OS are included in Supplementary Figs. S7–S10.

In similar methodology to the prediction of pathological response, a series of Cox regression models were constructed for RFS and OS. The variables cN-stage, differentiation of adenocarcinoma and DDIR status were included in all models because these three variables are available at the time of diagnosis, unlike pathological variables^7^. DDIR status was significant for RFS but not OS in the first model containing just three variables (cN-stage, differentiation of adenocarcinoma and DDIR status). (Table 5) The best performing model for both RFS and OS was model six, which contained cN-stage, differentiation of adenocarcinoma, DDIR status, and optimised 46.5% PET response threshold, which was independently significant for both RFS and OS (p = 0.012 and p = 0.047, respectively).

**Table 5:** Summary of results for each RFS and OS multi-variable Cox regression model.

Model	Variable	RFS					OS				
		HR	LCI	UCI	p-value	AIC	HR	LCI	UCI	p-value	AIC
1	cN-stage	1.59	0.97	2.60	0.066	430.57	1.45	0.86	2.46	0.163	361.77
	Differentiation	1.21	0.92	1.58	0.175		1.16	0.86	1.56	0.318	
	DDIR Status	0.45	0.20	1.00	0.049		0.49	0.20	1.16	0.105	
2	cN-stage	1.72	1.06	2.80	0.030	429.45	1.52	0.90	2.55	0.115	362.44
	Differentiation	1.21	0.92	1.58	0.176		1.16	0.86	1.55	0.338	
	DDIR Status	0.43	0.19	0.97	0.042		0.47	0.20	1.13	0.093	
	Baseline SUVmax	0.95	0.89	1.01	0.091		0.97	0.91	1.03	0.270	
3	cN-stage	1.61	0.98	2.63	0.058	432.17	1.47	0.87	2.49	0.150	363.47
	Differentiation	1.22	0.93	1.60	0.157		1.18	0.87	1.59	0.287	
	DDIR Status	0.47	0.21	1.07	0.071		0.51	0.21	1.24	0.136	
	Post-NACT SUVmax	1.03	0.95	1.11	0.510		1.02	0.95	1.10	0.563	
4	cN-stage	1.78	1.09	2.91	0.022	429.93	1.59	0.94	2.68	0.084	362.20
	Differentiation	1.23	0.94	1.62	0.135		1.18	0.88	1.59	0.270	
	DDIR Status	0.49	0.22	1.11	0.087		0.52	0.22	1.25	0.143	
	Change in SUVmax	0.99	0.99	1.00	0.089		0.99	0.99	1.00	0.192	
5	cN-stage	1.85	1.13	3.02	0.014	428.68	1.64	0.97	2.77	0.065	361.12
	Differentiation	1.21	0.92	1.59	0.180		1.16	0.86	1.57	0.316	
	DDIR Status	0.43	0.19	0.97	0.041		0.46	0.19	1.11	0.084	
	PET response at 35% threshold	0.55	0.31	0.98	0.044		0.59	0.31	1.10	0.098	
6	cN-stage	1.88	1.14	3.12	0.014	425.88	1.69	0.99	2.90	0.055	359.59
	Differentiation	1.19	0.90	1.57	0.214		1.14	0.85	1.54	0.384	
	DDIR Status	0.45	0.20	1.02	0.055		0.49	0.20	1.17	0.108	
	PET response at 46.5% threshold	0.47	0.26	0.85	0.012		0.51	0.26	0.99	0.047	

*HR* hazard ratio, *LCI* lower 95% confidence interval, *UCI* upper 95% confidence interval, *AIC* Akaike Information Criterion, *DDIR* DNA-damage immune response.

## Discussion

This study has demonstrated that PET metrics are predictive of pathological response and have additional value when combined with a novel pre-treatment DDIR assay. A model using both PET metrics and a DDIR assay improves the prediction of which patients are likely to have a pathological response. Post-treatment SUVmax and change in SUVmax were both independently predictive of pathological response and, when combined with a DDIR assay, demonstrated a greater predictive performance than DDIR alone. These findings add evidence to incorporating PET metrics into pre-treatment clinical decision tools and to re-stage OAC patients with PET-CT prior to surgery.

Pathological response is an important endpoint in oesophageal adenocarcinoma. Only 15% of patients have a meaningful benefit from neo-adjuvant chemotherapy, defined as Mandard TRG 1–2^15^. In this cohort, only 13 of 113 patients (11.5%) were classified as TRG 1 or 2. It is imperative that patient selection for radical curative treatment improves because despite aggressive treatment, the 2-year overall survival after neo-adjuvant chemotherapy and oesophagectomy ranges from 40 to 70%^7,18^ with a 20% recurrence rate^5^.

PET metrics were also associated with RFS and OS in patients with oesophageal adenocarcinoma treated with neo-adjuvant chemotherapy. Using a 35% reduction threshold, PET response was prognostically significant for both RFS and OS in univariable analysis. A series of multi-variable models were constructed using clinicopathological factors (cN-stage and degree of differentiation), PET metrics and DDIR status. Baseline SUVmax, post-treatment SUVmax and change in SUVmax were all included separately in multivariable models to assess the added value of PET metrics to predict pathological response. In addition, the optimised 46.5% SUVmax reduction threshold used to predict pathological response was independently significant for both RFS and OS in this cohort. However, these results must be validated in a new independent cohort prior to clinical adoption.

This work expanded on a study by Turkington et al.^7^ investigating a novel DDIR assay to predict pathological response prior to treatment initiation. The DDIR assay was originally developed in breast cancer and constitutes a 44-gene assay^19^ indicating constitutive activation of the cyclic GMP-AMP synthase (cGAS)/stimulator of interferon genes (STING) pathway in response to DNA damaging chemotherapy^20^. The DDIR assay includes immune checkpoint targets, such as programmed death ligand 1 (PD-L1)^21^, which are up-regulated by immune activation caused by infiltration of the tumour by T-lymphocytes. Up-regulation creates an inflammatory microenvironment associated with sensitivity to chemotherapy.

Whole genome sequencing in pre-treatment oesophageal adenocarcinoma biopsy samples has identified three distinct subtypes^6^. One sub-type, representing 20% of cases, had impaired DNA-damage repair status. These patients are likely to respond to DNA-damaging chemotherapy. Therefore, identification of this sub-group of patients prior to treatment initiation would be beneficial, because it may allow refinement of treatment approaches. The pre-treatment DDIR assay was strongly predictive of pathological response and can be performed using formalin-fixed material^7^. However, the diagnostic performance of the assay for pathological response could be improved further. We hypothesised that the addition of PET metrics to the DDIR assay could achieve this necessary improvement.

PET response is often defined as a 35% interval reduction in SUVmax. This threshold is based on work assessing early PET response in oesophageal adenocarcinoma to predict pathological response^11,22,23^. After 14 days, an early repeat PET-CT evaluated the change in SUVmax from baseline PET-CT and attempted to predict pathological response. In original work, Weber et al.^22^ demonstrated that the mean SUVmax of PET responding OAC (− 54% ± 17%) was significantly different from that of non-responding OAC (− 15% ± 21%). The optimal threshold value to differentiate these groups after one cycle was 35% SUVmax reduction, which resulted in a sensitivity of 93% and a specificity of 95%. This threshold was validated in subsequent work by this group, although the compelling diagnostic performance was not replicated. Ott et al.^23^ found the sensitivity and specificity of using these criteria were 80% and 78%, respectively. Lordick et al.^11^ found the sensitivity and specificity to be 100% and 72%, respectively. These diagnostic accuracy values were calculated against the reference standard pathological response criteria by Becker et al.^24^, defined as Becker classification 1 (< 10% tumour cells remaining).

Subsequently, authors have applied the 35% threshold to predict pathological response after completion of neo-adjuvant chemotherapy, when PET-CT is repeated approximately 3 months after staging. Results of studies show variable benefit^25^. Currently, no evidence exists to support the use of PET metrics to accurately predict pathological response. In our study, the 35% threshold did not show a significant association with pathological response, which suggests that alternative thresholds used to define metabolic PET response after completion of neo-adjuvant chemotherapy should be sought. However, the study identified an optimised threshold of 46.5% in this cohort of OAC patients. This threshold was significantly associated with pathological response, RFS and OS. These results are promising however they must be validated in external cohorts before being applied in clinical practice.

Findlay et al.^26^ investigated PET response between staging and post neo-adjuvant chemotherapy. The authors demonstrated that a larger reduction of SUVmax after treatment completion may be more predictive of pathological response. Whilst only a single-centre study, a SUVmax reduction of 77.8% performed better than the PERCIST threshold of 30%^27^ and the 35% threshold. Findlay et al. suggested using a more pragmatic threshold of 75% would result in a sensitivity of 73.6% and a specificity of 84.0%. Again, these results must be validated in external centres, but in combination with our results, suggest that a threshold above the commonly cited 35% threshold used after completion of neo-adjuvant treatment should be considered.

There are some limitations of the present study; this is a retrospective, single-centre study conducted over a 10-year period. These data are consistent and homogenous but require validation in a multi-centre study. Relatively few pathological responders were included in each DDIR status group. Early models of PET-CT scanners have inferior spatial resolution and signal-to-noise ratio compared to more modern systems, which may affect quantification of SUVmax, particularly for small tumours. The findings of this study must be validated in larger external cohorts using pre-defined PET response thresholds. The added value of PET metrics to the DDIR assay must also be tested in alternative neo-adjuvant therapy regimens including peri-operative FLOT (docetaxel, oxaliplatin, leucovorin, and 5-fluorouracil)^28^ and neo-adjuvant chemoradiotherapy^29^. Combining these biomarkers, whilst predictive of pathological response, does not inform the use of neo-adjuvant treatment.

In conclusion, this study has demonstrated the additional value of PET metrics, in combination with a novel transcriptomic DDIR assay, to predict pathological response in OAC patients treated with neo-adjuvant chemotherapy. Furthermore, an optimised SUVmax reduction threshold was calculated and was independently significant for RFS and OS. Combining imaging and molecular biomarkers could lead to improved stratification and precision therapeutic approaches in oesophageal adenocarcinoma.

## Supplementary Information

Supplementary Information.

## Data Availability

The datasets generated during and/or analysed during the current study are available in the Array Express repository, Accession Number E-MTAB-6969.

## Abbreviations

OAC: Oesophageal adenocarcinoma
DDIR: DNA-damage immune response
RFS: Recurrence-free survival
OS: Overall survival
PET: Positron emission tomography
TME: Tumour microenvironment
TRIPOD: Transparent reporting of a multivariable prediction model for individual prognosis or diagnosis
GI: Gastrointestinal
UICC: International Union for Cancer Control
TNM: Tumour node metastasis
ECX: Epirubicin, cisplatin and capecitabine
ECF: Epirubicin, cisplatin and 5-fluorouracil
TRG: Tumour regression grade
ROC: Receiver operator characteristic
CRM: Circumferential resection margin
cGAS: Cyclic GMP-AMP synthase
STING: Stimulator of interferon genes
PD-L1: Programmed death ligand 1
FLOT: Docetaxel, oxaliplatin, leucovorin, and 5-fluorouracil
