# Supplementary material for: A DNA-damage immune response assay combined with PET biomarkers predicts response to neo-adjuvant chemotherapy and survival in oesophageal adenocarcinoma
Source: Sci Rep. 2021 Jun 22;11:13061. doi: 10.1038/s41598-021-92545-w (PMC8219719; doi:10.1038/s41598-021-92545-w)
Supplement: Supplementary file 1 — Supplementary Information. [file 41598_2021_92545_MOESM1_ESM.docx]

**Supplementary Material**

**A DNA-Damage Immune Repair Assay Combined with PET Biomarkers Predicts Response to Neo-Adjuvant Chemotherapy and Survival in Oesophageal Adenocarcinoma**

Kieran Foley^1*^, Anita Lavery^2^, Eoin Napier^3^, David Campbell^3^, Martin M Eatock^2,3^, Richard D Kennedy^4^, Kevin M Bradley^5^, Richard C Turkington^2^

1 Velindre Cancer Centre, Cardiff, UK

2 Queen’s University Belfast, UK

3 Belfast Health and Social Care Trust, Belfast, UK

4 Almac Diagnostics, Craigavon, UK

5 Wales Research & Diagnostic Positron Emission Tomography Imaging Centre (PETIC), Cardiff University, UK

Supplementary Table S1. SUVmax statistics of PET-CT examinations.

| Median (Min, Max, IQR) | DDIR Negative | DDIR Positive | p-value |
| --- | --- | --- | --- |
|  |  |  |  |
| Baseline SUVmax | 9.30 (3.40, 39.50, 5.95) | 10.20 (3.10, 20.50, 7.00) | 0.40 |
|  |  |  |  |
| Post-chemotherapy SUVmax | 5.20 (0, 30.00, 3.13) | 3.60 (0, 15.80, 3.40) | 0.088 |
|  |  |  |  |
| Change in SUVmax (%) | 43.07 (-100.00, 100.00, 35.75) | 58.620 (-53.40, 100.00, 52.30) | 0.14 |


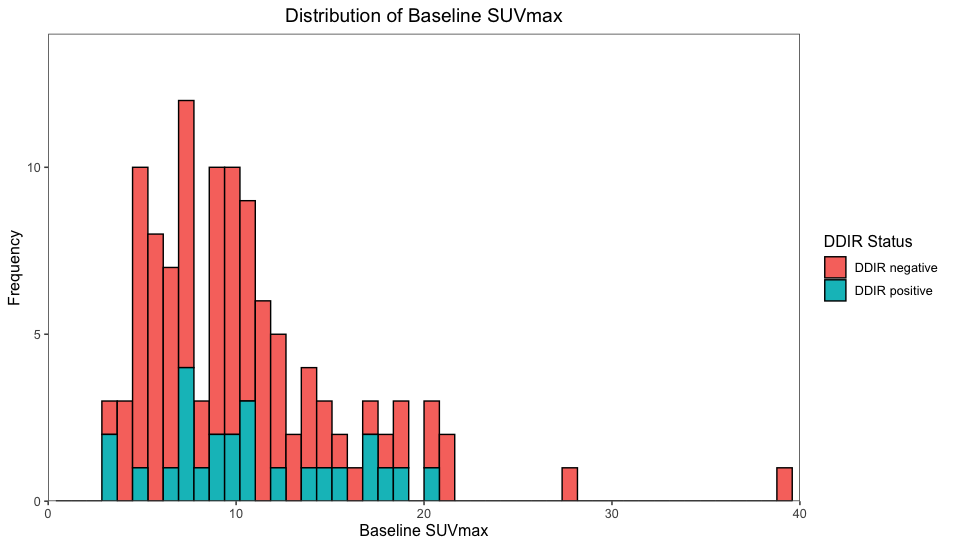


Supplementary Figure S1. Distribution of baseline SUVmax in patient cohort.


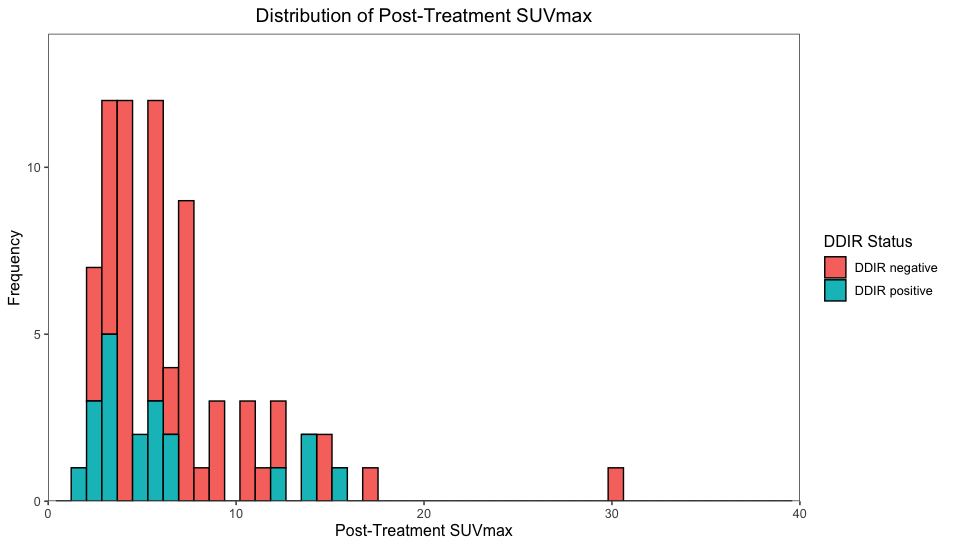


Supplementary Figure S2. Distribution of post-neo-adjuvant chemotherapy SUVmax in patient cohort.


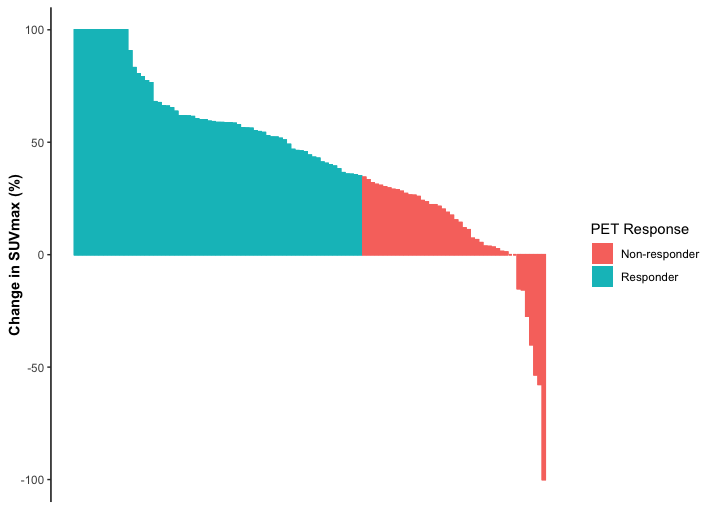


Supplementary Figure S3. Waterfall plot showing per-patient change in SUVmax.

Supplementary Table S2. Chi-square tests with Bonferroni adjustment for multiple comparisons between clinical variables

|  | Variable | Chi-square | df | Adjusted p-value |
| --- | --- | --- | --- | --- |
| DDIR Status | PET response | 0.01 | 1 | 1.00 |
|  | Chemotherapy type | 0.89 | 2 | 1.00 |
|  | cT-stage | 3.33 | 3 | 1.00 |
|  | cN-stage | 1.53 | 3 | 1.00 |
|  | pT-stage | 6.93 | 4 | 1.00 |
|  | pN-stage | 1.96 | 3 | 1.00 |
|  | Differentiation | 4.28 | 3 | 1.00 |
|  | LVI | 0.00 | 1 | 1.00 |
|  | CRM | 4.75 | 1 | 0.23 |
| Pathological Response | PET response | 7.61 | 1 | 0.05 |
|  | Chemotherapy type | 0.42 | 2 | 1.00 |
|  | cT-stage | 3.93 | 3 | 1.00 |
|  | cN-stage | 13.59 | 3 | 0.03 |
|  | pT-stage | 43.67 | 4 | <0.001 |
|  | pN-stage | 12.38 | 3 | 0.06 |
|  | Differentiation | 11.76 | 3 | 0.07 |
|  | LVI | 11.67 | 1 | 0.01 |
|  | CRM | 6.69 | 1 | 0.09 |
|  | DDIR Status | 0.20 | 1 | 1.00 |
| Recurrence | PET response | 0.04 | 1 | 1.00 |
|  | Chemotherapy type | 5.08 | 2 | 0.87 |
|  | cT-stage | 7.13 | 3 | 0.68 |
|  | cN-stage | 6.99 | 3 | 0.72 |
|  | pT-stage | 16.94 | 4 | 0.02 |
|  | pN-stage | 28.91 | 3 | <0.001 |
|  | Differentiation | 6.93 | 3 | 0.74 |
|  | LVI | 6.16 | 1 | 0.13 |
|  | CRM | 23.73 | 1 | <0.001 |
|  | DDIR Status | 2.60 | 1 | 1.00 |
|  | Pathological response | 6.40 | 1 | 0.11 |

LVI local vascular invasion; CRM circumferential resection margin

Supplementary Figure S4. Receiver operator characteristic curves for baseline SUVmax, post-NACT SUVmax and change in SUVmax association with pathological response.

Supplementary Table S3. Optimum SUVmax reduction thresholds for sensitivity and specificity.

|  | Sensitivity | Specificity | SUVmax reduction threshold (%) |
| --- | --- | --- | --- |
| **DDIR** |  |  |  |
| Baseline SUVmax | 0.32 | 0.83 | 13.95 |
| Post SUVmax | 0.56 | 0.78 | 3.70 |
| Change SUVmax | 0.52 | 0.73 | 58.61 |
| **Path Response** |  |  |  |
| Baseline SUVmax | 0.77 | 0.48 | 8.95 |
| Post SUVmax | 0.69 | 0.66 | 4.35 |
| Change SUVmax | 1.00 | 0.60 | 46.52 |
| **Recurrence** |  |  |  |
| Baseline SUVmax | 0.83 | 0.35 | 12.35 |
| Post SUVmax | 0.58 | 0.54 | 4.85 |
| Change SUVmax | 0.73 | 0.43 | 58.04 |

Supplementary Figure S5. Individual receiver operator characteristic curves for PET metrics associated with DDIR status, pathological response and recurrence.

Supplementary Table S4. Univariable analysis of clinicopathological variables and PET metrics to predict pathological response.

| Variable | Coefficient | SE | p-value | Adjusted p-value |
| --- | --- | --- | --- | --- |
| Gender | 0.51 | 0.65 | 0.428 | 1.000 |
| Age | 0.07 | 0.04 | 0.057 | 0.857 |
| Baseline SUVmax | 0.04 | 0.05 | 0.330 | 1.000 |
| Post-NACT SUVmax | -0.30 | 0.12 | 0.015 | 0.221 |
| Change in SUVmax | 0.04 | 0.01 | 0.002 | 0.029 |
| PET Response at 35% threshold | 18.11 | 1621.23 | 0.991 | 1.000 |
| PET Response at 46.5% threshold | 1.47 | 0.64 | 0.021 | 0.310 |
| cT-stage | 0.38 | 0.80 | 0.635 | 1.000 |
| cN-stage | 0.19 | 0.54 | 0.722 | 1.000 |
| pT-stage | -1.83 | 0.42 | <0.001 | <0.001 |
| pN-stage | -1.63 | 0.64 | 0.012 | 0.176 |
| Differentiation | -1.20 | 0.44 | 0.006 | 0.095 |
| LVI | -3.01 | 1.07 | 0.005 | 0.074 |
| CRM | -2.48 | 1.06 | 0.019 | 0.286 |
| DDIR Status | 0.51 | 0.65 | 0.428 | 1.000 |

SE standard error; LVI local vascular invasion; CRM circumferential resection margin; TRG tumour regression grade; DDIR DNA-damage immune response

Supplementary Table S5. Univariable analysis of clinicopathological variables and PET metrics for recurrence-free survival.

| Variable | HR | LCI | UCI | p-value | Adjusted p-value |
| --- | --- | --- | --- | --- | --- |
| Gender | 0.82 | 0.45 | 1.51 | 0.528 | 1.000 |
| Age | 1.01 | 0.98 | 1.03 | 0.605 | 1.000 |
| Baseline SUVmax | 0.96 | 0.91 | 1.01 | 0.087 | 1.000 |
| Post-NACT SUVmax | 1.04 | 0.99 | 1.10 | 0.134 | 1.000 |
| Change in SUVmax | 0.99 | 0.99 | 1.00 | 0.015 | 0.295 |
| PET Response at 35% threshold | 0.58 | 0.36 | 0.95 | 0.032 | 0.640 |
| PET Response at 46.5% threshold | 0.59 | 0.34 | 1.00 | 0.050 | 1.000 |
| cT-stage | 1.21 | 0.69 | 2.13 | 0.496 | 1.000 |
| cN-stage | 1.66 | 1.05 | 2.63 | 0.029 | 0.588 |
| pT-stage | 2.07 | 1.42 | 3.00 | <0.001 | 0.003 |
| pN-stage | 2.20 | 1.77 | 2.74 | <0.001 | <0.001 |
| Differentiation | 1.39 | 1.09 | 1.78 | 0.008 | 0.153 |
| LVI | 2.54 | 1.46 | 4.40 | 0.001 | 0.018 |
| CRM | 4.85 | 2.82 | 8.34 | <0.001 | <0.001 |
| Total Resected Nodes | 0.99 | 0.96 | 1.02 | 0.539 | 1.000 |
| Total Positive Nodes | 1.19 | 1.14 | 1.24 | <0.001 | <0.001 |
| Positive Node Ratio | 1.04 | 1.03 | 1.05 | <0.001 | <0.001 |
| TRG | 1.60 | 1.20 | 2.12 | 0.001 | 0.026 |
| Pathological Response | 0.16 | 0.04 | 0.67 | 0.012 | 0.232 |
| DDIR Status | 0.52 | 0.26 | 1.02 | 0.058 | 1.000 |

HR hazard ratio; LCI lower 95% confidence interval; UCI upper 95% confidence interval; AIC Akaike Information Criterion; LVI local vascular invasion; CRM circumferential resection margin; TRG tumour regression grade; DDIR DNA-damage immune response

Supplementary Table S6. Univariable analysis of clinicopathological variables and PET metrics for overall survival.

| Variable | HR | LCI | UCI | p-value | Adjusted p-value |
| --- | --- | --- | --- | --- | --- |
| Gender | 0.69 | 0.35 | 1.38 | 0.298 | 1.000 |
| Age | 1.01 | 0.98 | 1.04 | 0.391 | 1.000 |
| Baseline SUVmax | 0.96 | 0.91 | 1.02 | 0.162 | 1.000 |
| Post-NACT SUVmax | 1.04 | 0.98 | 1.09 | 0.197 | 1.000 |
| Change in SUVmax | 0.99 | 0.99 | 1.00 | 0.024 | 0.509 |
| PET Response at 35% threshold | 0.55 | 0.33 | 0.94 | 0.030 | 0.631 |
| PET Response at 46.5% threshold | 0.61 | 0.34 | 1.10 | 0.102 | 1.000 |
| cT-stage | 1.44 | 0.75 | 2.76 | 0.270 | 1.000 |
| cN-stage | 1.50 | 0.91 | 2.47 | 0.108 | 1.000 |
| pT-stage | 2.30 | 1.51 | 3.51 | <0.001 | 0.002 |
| pN-stage | 2.26 | 1.78 | 2.86 | <0.001 | <0.001 |
| Differentiation | 1.37 | 1.05 | 1.78 | 0.020 | 0.429 |
| LVI | 4.22 | 2.12 | 8.40 | <0.001 | 0.001 |
| CRM | 5.02 | 2.78 | 9.05 | <0.001 | <0.001 |
| Total Resected Nodes | 1.00 | 0.97 | 1.03 | 0.923 | 1.000 |
| Total Positive Nodes | 1.16 | 1.11 | 1.21 | <0.001 | <0.001 |
| Positive Node Ratio | 1.04 | 1.03 | 1.05 | <0.001 | <0.001 |
| TRG | 1.57 | 1.15 | 2.14 | 0.004 | 0.093 |
| Pathological Response | NA | NA | NA | 0.995 | 1.000 |
| DDIR Status | 0.58 | 0.28 | 1.19 | 0.139 | 1.000 |
| Recurrence | 12.48 | 5.31 | 29.31 | <0.001 | <0.001 |

HR hazard ratio; LCI lower 95% confidence interval; UCI upper 95% confidence interval; AIC Akaike Information Criterion; LVI local vascular invasion; CRM circumferential resection margin; TRG tumour regression grade; DDIR DNA-damage immune response


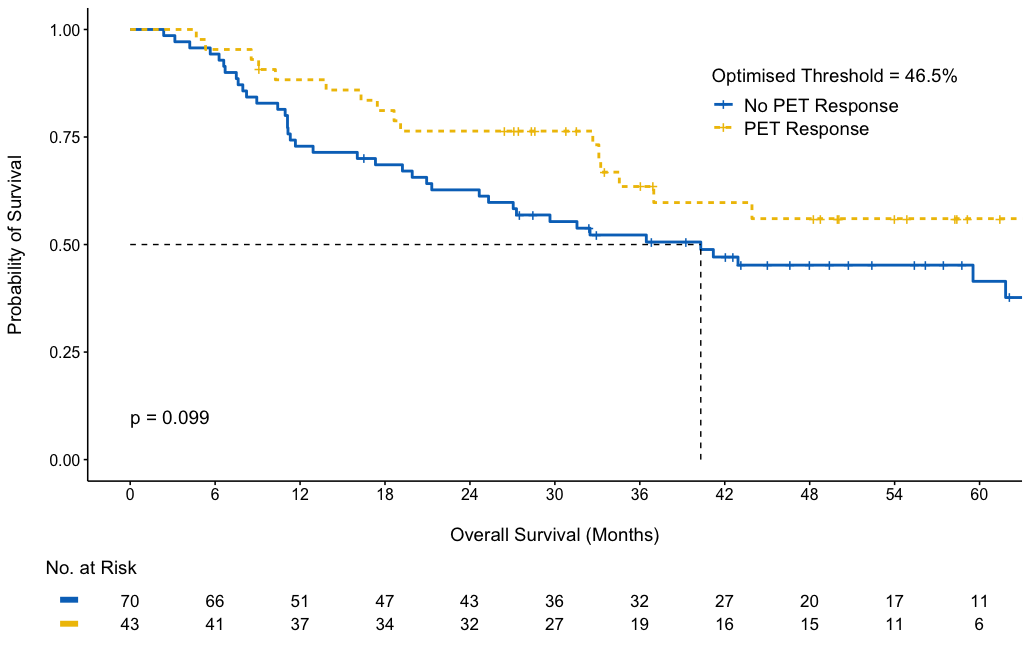


Supplementary Figure S6. Overall survival difference between pathological responders and non-responders using the optimised threshold of 46.5% reduction in SUVmax.


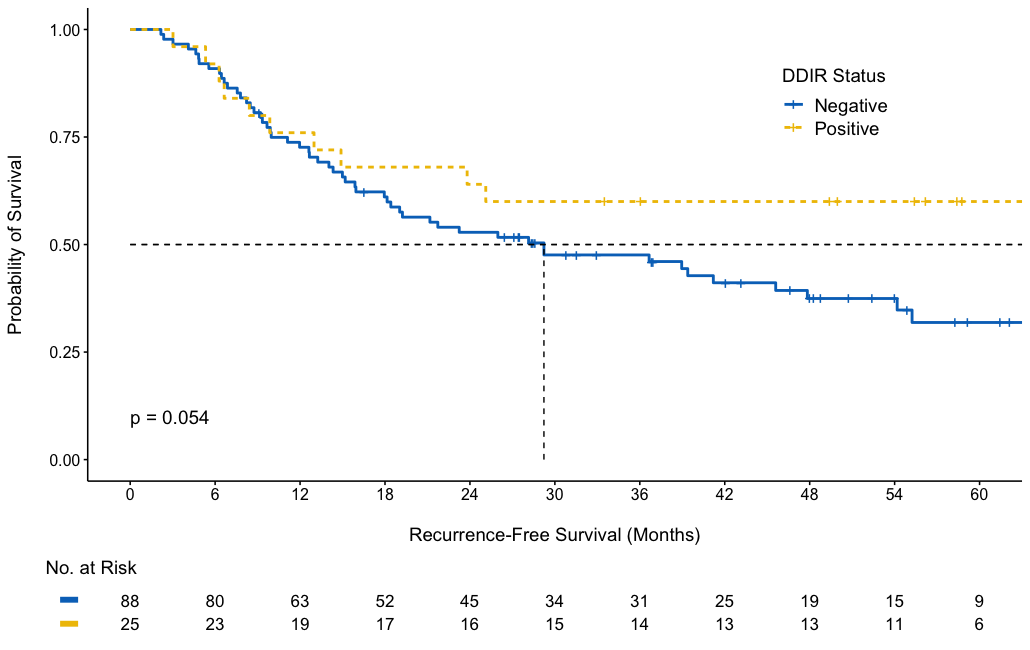


Supplementary Figure S7. Recurrence-free survival difference between DDIR status using the 35% threshold reduction in SUVmax.


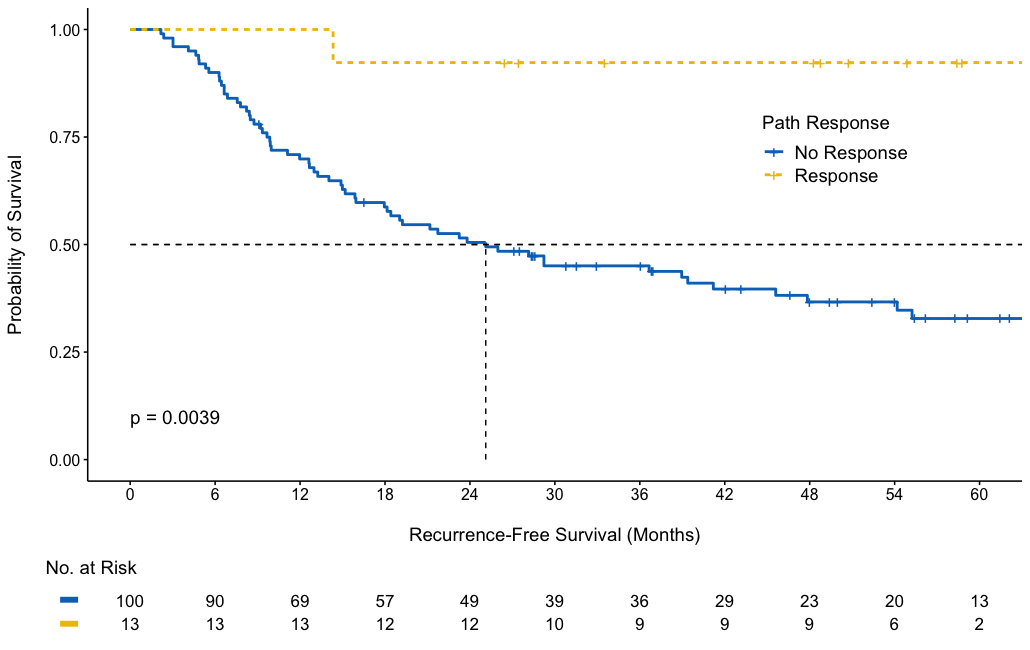


Supplementary Figure S8. Recurrence-free survival difference between pathological responders and non-responders using the 35% threshold reduction in SUVmax.


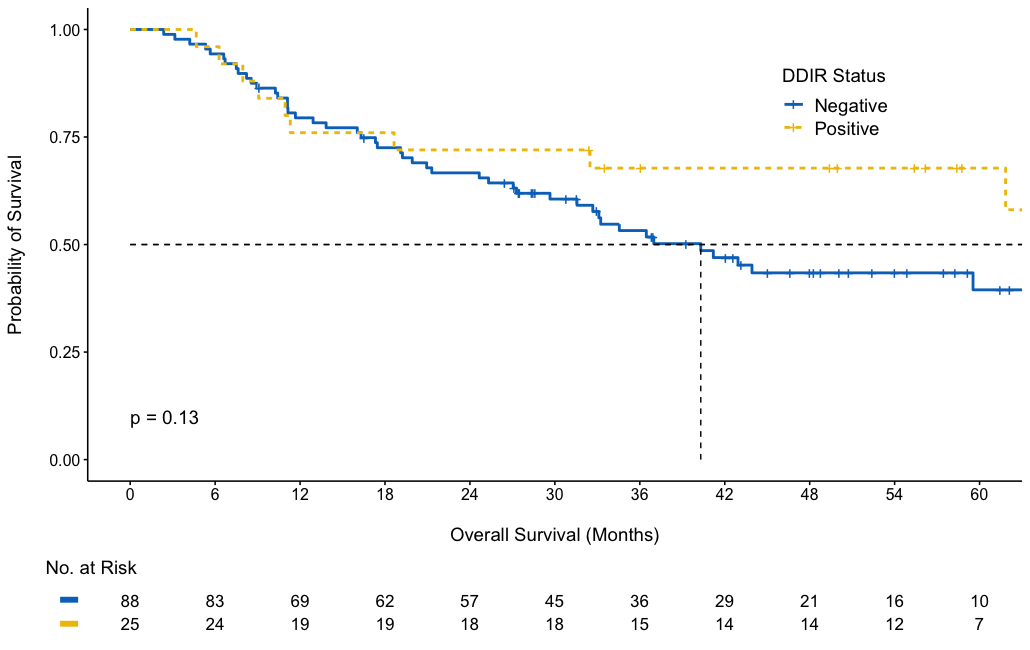


Supplementary Figure S9. Overall survival difference between DDIR status using the 35% threshold reduction in SUVmax.


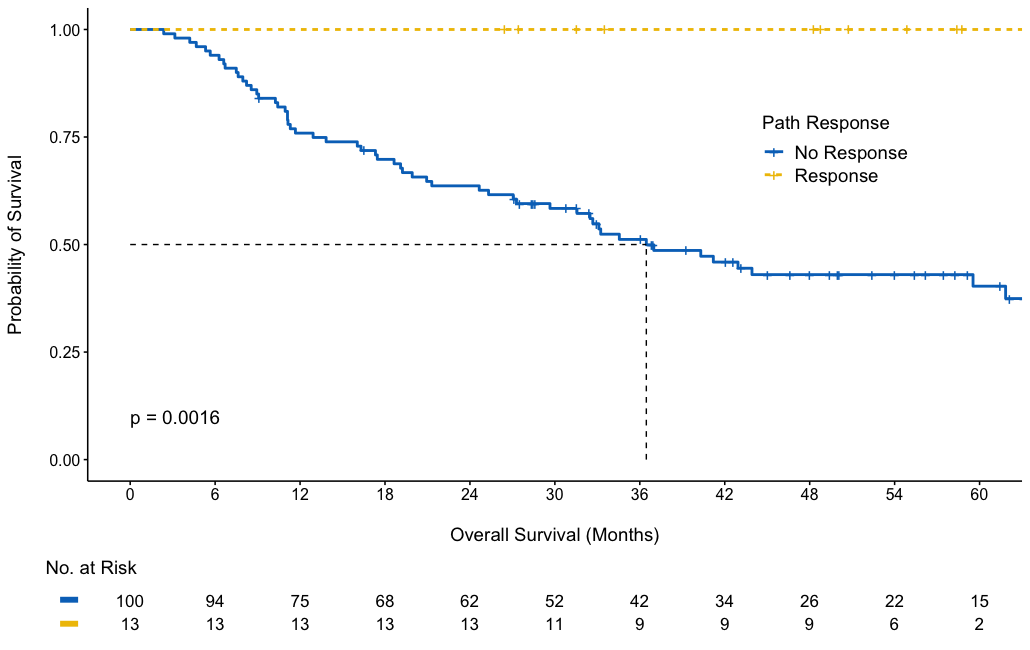


Supplementary Figure S10. Overall survival difference between pathological responders and non-responders using the 35% threshold reduction in SUVmax.
